# Supplementary material for: Prediction of Antibody Viscosity from Dilute Solution Measurements
Source: Antibodies (Basel). 2023 Dec 1;12(4):78. doi: 10.3390/antib12040078 (PMC10740665; doi:10.3390/antib12040078)
Supplement: Supplementary file 1 [file antibodies-12-00078-s001.zip › antibodies-2673439-supplementary.pdf]

Supplementary Materials

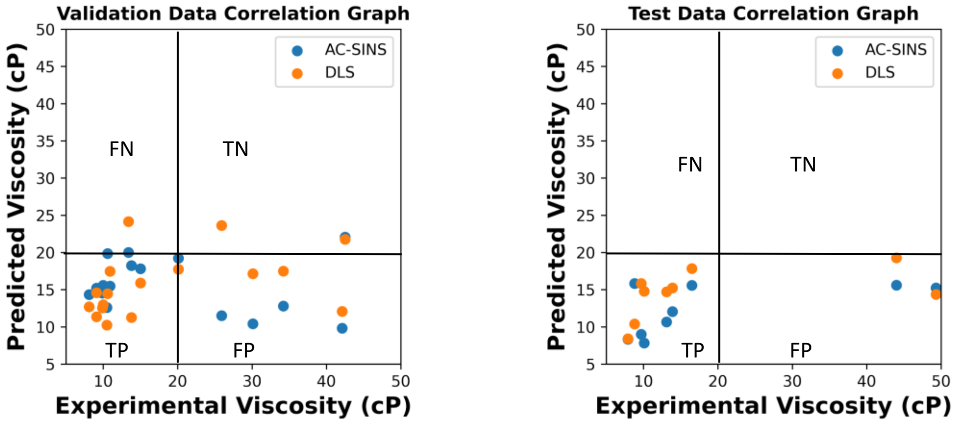

Figure S1: Our physics-based model generally predicts antibody viscosity accurately, with the exception of a few outliers. To differentiate between good and bad molecules, we've set a cutoff value of 20 cP. Predicted viscosities are evaluated at the experimental concentrations, and notably, the majority of outliers fall into the category of False Positives. These outliers, classified incorrectly by our model, can be isolated by applying a revised cutoff line in Figure 5. In the context of this analysis, abbreviations are used as follows: TP (True Positive), TN (True Negative), FP (False Positive), and FN (False Negative). Blue dots represent the model-predicted viscosity values from AC-SINS measurements (D_{np}), while orange dots represent the model-predicted viscosity values from DLS measurements (k_D), both corresponding to the concentrations at which actual viscosity values are measured.
